# Nutritional Risk, Weight Loss, and Inflammatory Profiles During Autologous Stem Cell Transplantation: Clinical Correlates in Patients with Lymphoma and Multiple Myeloma

**DOI:** 10.3390/nu18213636

**Published:** 2026-10-31

**Authors:** Berrin Balik, Defne Beybolat, Osama Alabdullah, Hande Erkaya, Nur I. Genc, Barıs Gerenli, Elif Ertas, Zeynep Atceken, Tugce Aytulu, Taner Tan, Khayala Mammadova, Emre Osmanbasoglu, Umit Barbaros Ure, Olga Meltem Akay, Sinem Civriz Bozdag

**Affiliations:** 1 Department of Hematology, Koc University Hospital, Istanbul 34010, Türkiye; tanertan1989@gmail.com (T.T.); kmammadova@kuh.ku.edu.tr (K.M.); eosmanbasoglu@yahoo.com (E.O.); uure@ku.edu.tr (U.B.U.); olakay@ku.edu.tr (O.M.A.); sbozdag@kuh.ku.edu.tr (S.C.B.); 2 School of Medicine, Koc University, Istanbul 34450, Türkiye; dbeybolat19@ku.edu.tr (D.B.); oabdullah20@ku.edu.tr (O.A.); 3 Department of Nutrition and Dietetics, Koc University Hospital, Istanbul 34010, Türkiye; herkaya@kuh.ku.edu.tr; 4 Department of Internal Medicine, Koc University Hospital, Istanbul 34010, Türkiye; ngenc@kuh.ku.edu.tr (N.I.G.); bgerenli@kuh.ku.edu.tr (B.G.); 5 Department of Biostatistics and Medical Informatics, Faculty of Medicine, Atlas University, Istanbul 34408, Türkiye; elif.ertas@atlas.edu.tr; 6 Department of Radiology, Koc University Hospital, Istanbul 34010, Türkiye; zatceken@kuh.ku.edu.tr; 7 Department of Nutrition and Dietetics, American Hospital, Istanbul 34365, Türkiye; tugcea@amerikanhastanesi.org

**Keywords:** autologous stem cell transplantation, nutritional risk, NRS-2002, measured body-weight change, lymphoma, multiple myeloma

## Abstract

Background/Objectives: Nutritional deterioration during autologous stem cell transplantation (ASCT) is driven by conditioning-related gastrointestinal toxicity, reduced oral intake, inflammatory stress, and catabolism. We evaluated the relationships among NRS-2002 nutritional risk, admission-to-discharge change in measured body weight, nutrition-impact symptoms, inflammatory markers, and clinical characteristics in patients with lymphoma and multiple myeloma. Methods: This retrospective, single-center observational cohort included 131 adults undergoing ASCT for lymphoma (*n* = 74) or multiple myeloma (*n* = 57) between 2022 and 2024. Nutritional risk was assessed at admission using NRS-2002, and measured body-weight change, albumin, C-reactive protein (CRP), neutrophil-to-lymphocyte ratio (NLR), nutritional support, nutrition-impact symptoms, and length of stay were evaluated. A ≥5% reduction in measured body weight was used as a pragmatic exploratory threshold, not as a diagnostic malnutrition criterion. Results: High nutritional risk (NRS-2002 ≥ 3) was present in 33/131 patients (25%), while 118/131 (90%) had a lower measured discharge weight and 53/131 (40%) met the ≥5% threshold. NRS-2002 was not significantly associated with CRP, albumin, NLR, percentage measured body-weight reduction, or length of stay. In exploratory multivariable analysis (complete-case *n* = 92; 37 events), multiple myeloma was associated with lower odds of meeting the threshold than lymphoma (OR, 0.20; 95% CI, 0.07–0.56; *p* = 0.002), whereas higher admission BMI was associated with higher odds (OR, 1.12 per kg/m^2^; 95% CI, 1.01–1.24; *p* = 0.03). A continuous-outcome model also showed less measured body-weight reduction in multiple myeloma (β, −1.9 percentage points; *p* = 0.005), while the BMI association was borderline (*p* = 0.051). Conclusions: Reductions in measured body weight and nutrition-impact symptoms were common during ASCT. Because detailed fluid-related data were unavailable, measured weight change cannot be assumed to represent tissue loss. Admission NRS-2002 screening and subsequent acute weight change are related but distinct assessments. The diagnosis association may reflect unmeasured transplant-related differences, and the BMI association was not robust in sensitivity analyses.

## 1. Introduction

Autologous stem cell transplantation (ASCT) remains an important treatment strategy for transplant-eligible patients with multiple myeloma and for selected patients with relapsed or high-risk lymphoma. The transplant period is characterized by high-dose conditioning, mucosal injury, gastrointestinal symptoms, reduced oral intake, inflammatory stress, and periods of reduced physical activity. Together, these factors can contribute to acute nutritional deterioration. Contemporary cancer nutrition guidance therefore recommends systematic nutritional screening and repeated assessment during anticancer therapy [1].

Nutritional Risk Screening 2002 (NRS-2002) is a widely used hospital screening instrument that combines impaired nutritional status with disease severity and identifies patients at nutritional risk using a score of ≥3 [2]. Nutritional vulnerability is particularly relevant in hematopoietic stem cell transplantation (HSCT). Previous studies have linked pre-transplant malnutrition risk with mortality after HSCT, while nutritional support strategies have been investigated specifically in autologous transplant populations [3,4]. In a recent HSCT-candidate cohort, NRS-2002-defined nutritional risk was associated with lower BMI and albumin and higher inflammatory markers, emphasizing the interaction between nutritional and inflammatory phenotypes [5].

Systemic inflammation may contribute to nutritional deterioration through increased catabolism, altered protein metabolism, and reduced appetite. CRP and serum albumin are readily available laboratory variables influenced by systemic inflammation, illness severity, fluid status, hepatic synthesis, and treatment-related stress [5]. At the same time, the present study also evaluated NLR as a marker of systemic inflammatory activity. Evaluating these measures alongside NRS-2002 risk and subsequent measured body-weight change may help clarify whether conventional laboratory measures are associated with nutritional changes during ASCT.

Recent evidence has also demonstrated the clinical relevance of nutritional status in this population, with malnutrition at hospital admission associated with increased risks of intensive care unit admission and in-hospital mortality [6].

The primary outcome was percentage change in measured body weight from admission to discharge. The primary objective was to characterize nutritional vulnerability during ASCT by examining the relationships among NRS-2002 risk, measured body-weight change, nutrition-impact symptoms, inflammatory markers, and clinical characteristics. Secondary, exploratory outcomes were any reduction in measured body weight, meeting the ≥5% threshold, correlations with continuous measured body-weight change, and factors associated with meeting the threshold.

## 2. Methods

### 2.1. Study Design and Population

We conducted a retrospective, single-center observational cohort study of adult patients with lymphoma or multiple myeloma who underwent autologous stem cell transplantation (ASCT) at Koç University Hospital, Istanbul, Türkiye, between 2022 and 2024. All eligible patients who underwent ASCT during the study period were included. No otherwise eligible patients were excluded from the primary cohort. The study size was determined by the number of eligible patients undergoing ASCT during the predefined study period; no formal sample-size calculation was performed. The final cohort comprised 131 patients, including 74 patients with lymphoma and 57 with multiple myeloma.

Demographic, anthropometric, laboratory, nutritional, and symptom-related data were retrospectively collected from the available clinical records. Patients were eligible if they were aged ≥18 years, had a diagnosis of lymphoma or multiple myeloma, and underwent ASCT during the study period.

**Figure 1:**
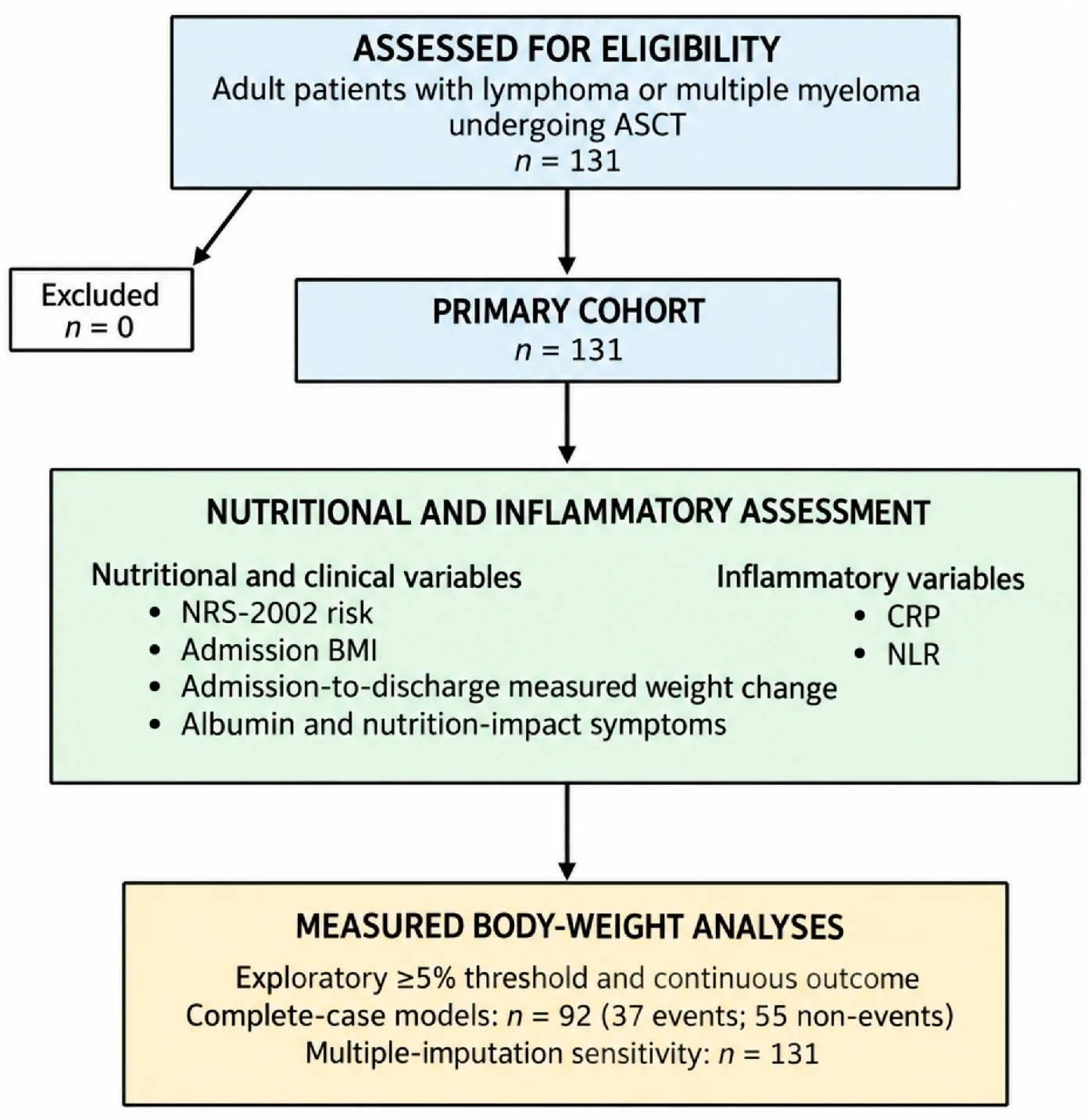
Study flow diagram. The primary cohort included 131 patients undergoing autologous stem cell transplantation. The complete-case multivariable model included 92 patients (37 events and 55 non-events); the multiple-imputation sensitivity analysis included all 131 patients. Denominators varied according to data availability.

The study was reported in accordance with the Strengthening the Reporting of Observational Studies in Epidemiology (STROBE) guidelines. Figure 1 summarizes the study flow.

### 2.2. Nutritional and Clinical Variables

Nutritional risk was assessed at admission to the transplant unit using Nutritional Risk Screening 2002 (NRS-2002), with a score of ≥3 defining high nutritional risk. Body mass index (BMI) was calculated from recorded admission height and weight, and obesity was defined as BMI ≥ 30 kg/m^2^. NRS-2002 was treated as an admission screening measure rather than a tool developed specifically to predict acute weight change.

The primary outcome was admission-to-discharge percentage change in measured body weight, calculated as [(admission weight − discharge weight)/admission weight] × 100; positive values indicate a lower measured discharge weight. Weights were obtained during routine care, and a study-specific protocol for time of day, clothing, fasting status, relation to intravenous fluids, or scale calibration could not be verified. The median interval between measurements was 24 days (IQR, 20–29). Edema or fluid overload, cumulative fluid balance, diuretic use, hydration status, and contemporaneous renal function were not available for adjustment. A ≥5% reduction was selected as a pragmatic exploratory threshold.

Laboratory variables included albumin (g/L), C-reactive protein (CRP; mg/L), and neutrophil-to-lymphocyte ratio (NLR) measured within ±10 days of the transplant admission, as available. Albumin was considered a biochemical variable influenced by inflammation, fluid status, hepatic synthesis, and treatment-related stress rather than a direct nutritional marker. Nutritional support provided during hospitalization was recorded, including oral nutritional supplement (ONS) and total parenteral nutrition (TPN) use. Timing and indications relative to symptoms and weight change were not consistently available; ONS and TPN were therefore treated as descriptive supportive-care variables. Nutrition-impact symptoms recorded during the transplant admission included dysphagia, nausea or odor sensitivity, mucositis, diarrhea, abdominal pain, and anorexia. Length of hospital stay was also recorded.

### 2.3. Statistical Analysis

Continuous variables were summarized as mean ± standard deviation or median with interquartile range (IQR), depending on distribution, and categorical variables as n/N (%). Continuous variables were compared between groups using Student’s *t*-test or the Mann–Whitney U test, as appropriate. Categorical variables were compared using Pearson’s chi-square test or Fisher’s exact test.

Spearman’s rank correlation was used to evaluate associations of NRS-2002 score with albumin, C-reactive protein (CRP), neutrophil-to-lymphocyte ratio (NLR), percentage measured body-weight reduction, and length of hospital stay.

Candidate covariates were selected based on clinical relevance and availability in the retrospective dataset. Sex was not prespecified and was omitted from the primary model because the event count constrained model complexity; it was included in a sensitivity model. ONS and TPN were excluded because their temporal order relative to symptoms and weight change was uncertain.

Multivariable logistic regression was used to explore factors associated with meeting the ≥5% measured body-weight reduction threshold. The model included age, diagnosis, NRS-2002 ≥ 3, admission BMI, albumin, CRP, diarrhea, and anorexia. Odds ratios (ORs) with 95% confidence intervals (CIs) were reported. The complete-case model included 92 patients (37 events and 55 non-events; 4.6 events per predictor). Sensitivity analyses used Firth penalization, a parsimonious model (age, diagnosis, NRS-2002, and BMI), and the same model with sex. Percentage measured body-weight reduction was also analyzed continuously using multivariable linear regression with HC3 robust standard errors.

Analyses were performed using available data for each variable, and variable-level missingness is reported in Table 1. As a sensitivity analysis under a missing-at-random assumption, 20 datasets were generated by chained equations and estimates were pooled using Rubin’s rules. CRP was log-transformed for imputation. Model performance was described using the area under the receiver operating characteristic curve (AUC), Brier score, Hosmer–Lemeshow test, and variance-inflation factors (VIFs). All tests were two-sided; *p* < 0.05 was used descriptively, and no adjustment for multiple comparisons was applied. Original analyses were performed in IBM SPSS Statistics version 25.0; sensitivity analyses were performed in Python 3.12.

## 3. Results

### 3.1. Cohort Characteristics and Nutritional Burden

The study included 131 patients: 74 with lymphoma and 57 with multiple myeloma. The mean age was 54 ± 14 years, 65/131 (50%) were female, and the median length of hospital stay—and interval between admission and discharge weight measurements—was 24 days (IQR, 20–29). Patients with multiple myeloma were older and had a higher admission BMI, while greater measured body-weight reduction was observed in the lymphoma group (Table 2).

High nutritional risk (NRS-2002 ≥ 3) was present in 33/131 patients (25%). Scores were 2 in 98 patients (75%), 3 in 22 (17%), 4 in 4 (3%), and 5 in 7 (5%). A lower measured discharge weight was observed in 118/131 patients (90%), while 53/131 (40%) met the ≥5% threshold. Median measured body-weight reduction was 4% (IQR, 2–6; range, −11% to 15%).

**Figure 2:**
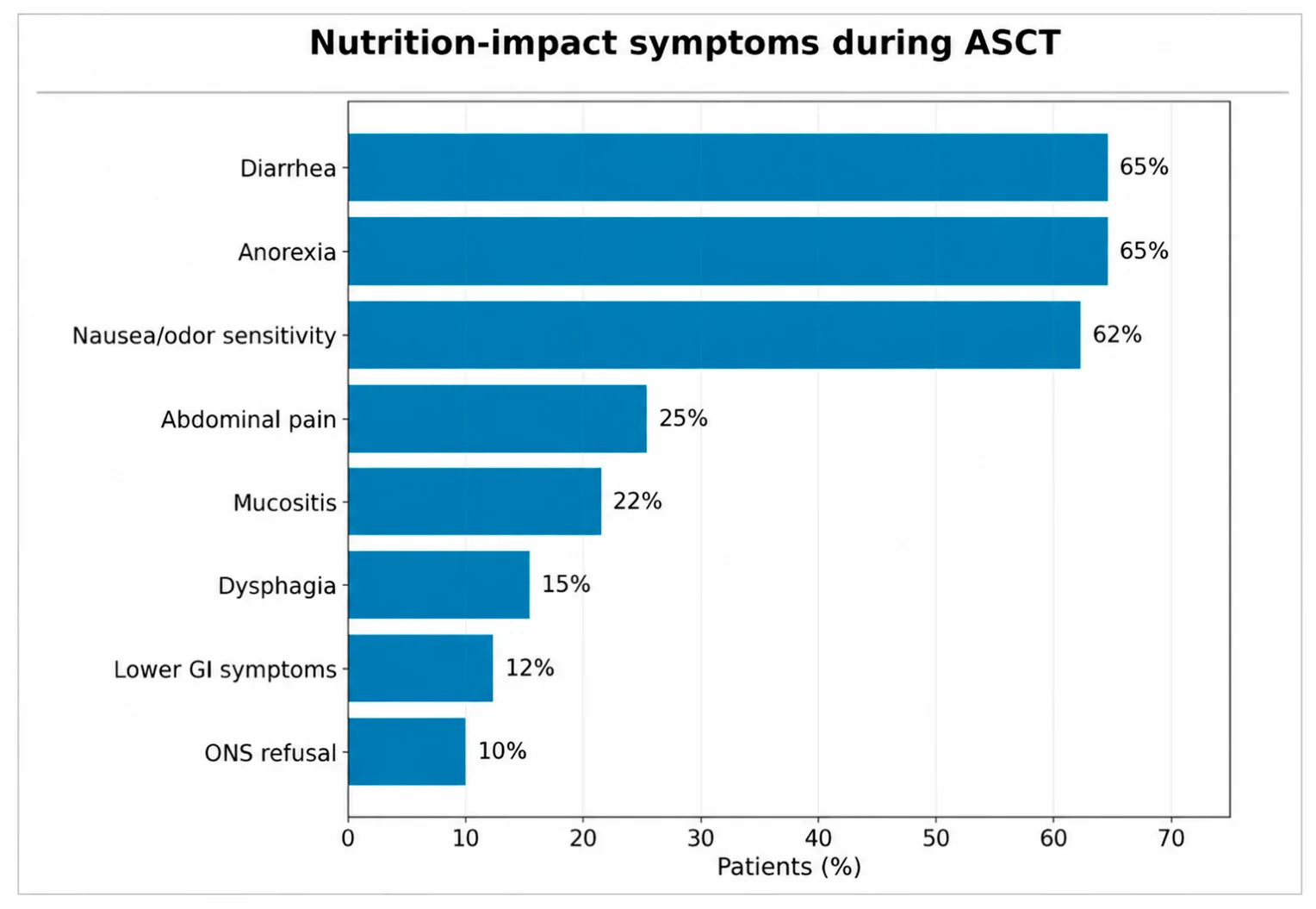
Prevalence of selected nutrition-impact symptoms during the ASCT hospitalization. Percentages are based on 130 evaluable patients for symptom variables.

Diarrhea and anorexia were the most common nutrition-impact symptoms, each affecting 84/130 patients (65%), followed by nausea or odor sensitivity in 81/130 (62%) (Figure 2). Oral nutritional supplements were used in 63/130 patients (48%), and total parenteral nutrition was used in 67/129 (52%). Because timing and indications were not consistently available, these support variables are descriptive and may be subject to confounding by indication.

### 3.2. Nutritional Risk and Inflammatory Profile

**Figure 3:**
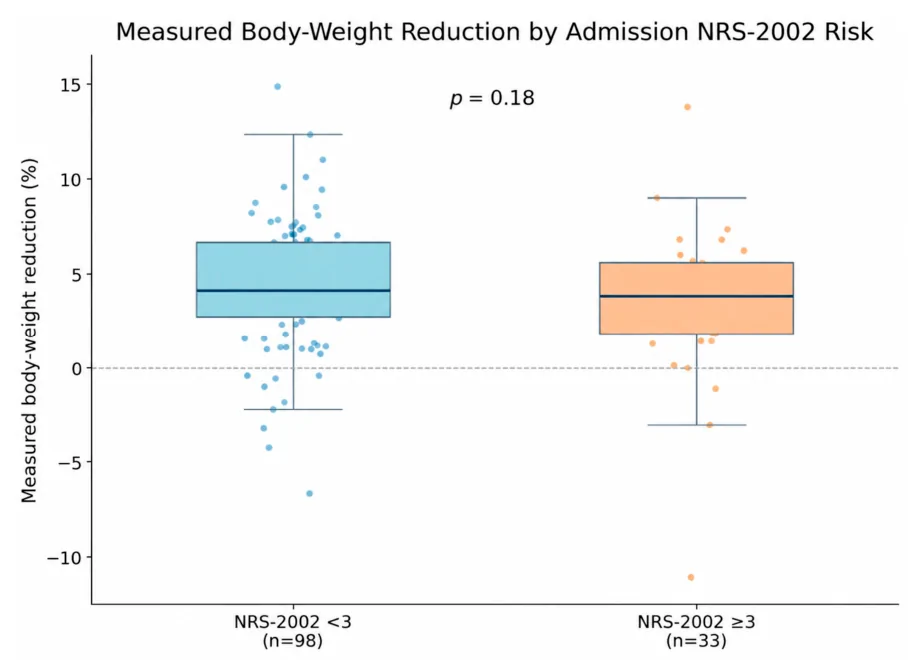
Distribution of continuous admission-to-discharge measured body-weight reduction by admission NRS-2002 risk category. Positive values indicate a lower measured discharge weight. Boxes show medians and interquartile ranges; points represent individual patients. The between-group comparison was not significant (*p* = 0.18).

NRS-2002 score was not significantly correlated with albumin (Spearman r = −0.10, *p* = 0.28), CRP (r = 0.16, *p* = 0.11), NLR (r = −0.02, *p* = 0.85), percentage measured body-weight reduction (r = −0.11, *p* = 0.19), or length of hospital stay (r = −0.08, *p* = 0.38). These findings are inconclusive rather than being evidence of no clinically meaningful association. The distribution of measured body-weight reduction was also comparable between the two NRS-2002 risk categories (Figure 3; *p* = 0.18).

Laboratory markers were also similar across NRS-2002 risk groups. Median albumin was 39.3 g/L (IQR, 36.9–41.1) in patients with NRS-2002 ≥ 3 and 40.1 g/L (IQR, 37.6–41.8) in those with NRS-2002 < 3 (*p* = 0.33). Median CRP was 3.3 (IQR, 1.4–8.6) versus 2.2 (IQR, 1.0–5.1; *p* = 0.14), and median NLR was 16 (IQR, 10.7–32) versus 19 (IQR, 10–36.5; *p* = 0.84), respectively. Rates of diarrhea, anorexia, and TPN use were similar between the NRS-2002 risk groups (Table 3).

### 3.3. Clinical Correlates of the ≥5% Measured Body-Weight Reduction Threshold

A total of 53/131 patients (40%) met the ≥5% measured body-weight reduction threshold. The threshold was met more often in patients with lymphoma than in those with multiple myeloma (38/74 [51%] vs. 15/57 [26%]; *p* = 0.004). Patients meeting the threshold were younger (median, 55 vs. 61 years; *p* = 0.01) and had a higher admission BMI (median, 27.6 vs. 26.0 kg/m^2^; *p* = 0.04). NRS-2002 category, albumin, CRP, NLR, ONS or TPN use, mucositis, diarrhea, and anorexia were not significant in univariable comparisons.

In exploratory multivariable logistic regression (complete-case *n* = 92; 37 events), multiple myeloma was associated with lower odds of meeting the ≥5% threshold than lymphoma (OR, 0.20; 95% CI, 0.07–0.56; *p* = 0.002), and higher admission BMI was associated with higher odds (OR, 1.12 per kg/m^2^; 95% CI, 1.01–1.24; *p* = 0.03). The age estimate was borderline (OR, 0.95; 95% CI, 0.91–1.00; *p* = 0.046) and was not emphasized. NRS-2002 ≥ 3, albumin, CRP, diarrhea, and anorexia were not independently associated with the threshold (Table 4).

Firth estimates were similar for diagnosis (OR, 0.23; 95% CI, 0.08–0.63; *p* = 0.005) and BMI (OR, 1.11; 95% CI, 1.01–1.22; *p* = 0.04). In the parsimonious full-cohort model, diagnosis remained associated with the threshold (OR, 0.36; 95% CI, 0.16–0.81; *p* = 0.01), whereas BMI did not (*p* = 0.09); adding sex gave similar results, and sex was not significant (*p* = 0.16). After multiple imputation, diagnosis remained associated (OR, 0.30; 95% CI, 0.13–0.71; *p* = 0.006) but BMI did not (*p* = 0.11). In the continuous complete-case model, multiple myeloma was associated with 1.9 percentage points less measured body-weight reduction (95% CI, −3.1 to −0.56; *p* = 0.005); BMI was borderline (*p* = 0.051) and NRS-2002 ≥ 3 was not significant (*p* = 0.74) (Table 5). The multiple-imputation continuous estimate for diagnosis was attenuated (*p* = 0.06).

## 4. Discussion

In this real-world ASCT cohort, reductions in measured body weight and nutrition-impact symptoms were common. Previous HSCT studies have similarly documented nutritional deterioration during transplantation [7,8]. Nearly nine in ten patients had a lower measured discharge weight, approximately 40% met the ≥5% threshold, and diarrhea and anorexia each affected almost two-thirds of the cohort. Approximately half received ONS or TPN, consistent with reports of frequent and variable nutrition-support use across HSCT practice [9,10]. These findings describe scale-weight change over a median of 24 days; intravenous fluids, diuresis, edema, gastrointestinal losses, renal function, and supportive treatments may also affect short-term body weight.

NRS-2002 remains an established screening tool for hospitalized patients [2,11,12], and pre-transplant nutritional risk has been associated with adverse outcomes in previous HSCT cohorts [3,13]. In the present cohort, NRS-2002 was not significantly associated with measured body-weight reduction. However, NRS-2002 was developed to identify nutritional risk at admission, not specifically to predict acute admission-to-discharge weight change. The wide confidence interval for NRS-2002 ≥ 3 (OR, 1.09; 95% CI, 0.34–3.47) makes this result inconclusive. Repeated assessment may therefore complement, rather than replace, baseline screening during ASCT.

Conventional inflammatory and biochemical markers showed limited concordance with baseline nutritional risk. CRP, NLR, and albumin were not significantly correlated with NRS-2002 score, and their distributions were similar between NRS-2002 risk groups. This differs from Zahedi et al., who reported associations between NRS-2002-defined nutritional risk and biochemical markers among HSCT candidates [5]. Differences in patient populations, the broad ±10-day laboratory window, transplant characteristics, and patterns of baseline inflammatory activity may partly explain this discrepancy. NRS-2002 assesses nutritional risk based on impaired nutritional status and disease severity [2]. In contrast, serum albumin is influenced by systemic inflammation, fluid status, hepatic synthesis, and treatment-related stress and is not a direct nutritional marker.

The high frequency of nutrition-impact symptoms provides an important clinical context for the measured weight changes. Diarrhea, anorexia, and nausea or odor sensitivity affected most patients, while approximately half received ONS or TPN. The timing of support relative to symptoms and weight change was unavailable, so confounding by indication is likely; ONS and TPN were treated as descriptive variables. Weight change during ASCT is likely multifactorial [4,13,14,15,16]. This exploratory ≥ 5% threshold should not be interpreted as a validated short-term malnutrition criterion because established diagnostic frameworks assess weight loss over longer defined intervals [17]. Current ESPEN guidance emphasizes repeated nutritional assessment and individualized intervention throughout cancer treatment [1].

In the exploratory multivariable analysis, diagnosis and admission BMI were associated with the ≥5% threshold. The diagnosis association was similar in Firth, parsimonious, sex-adjusted, and multiple-imputation models, and multiple myeloma was also associated with less reduction in the complete-case continuous model. Nevertheless, this may reflect unmeasured treatment differences rather than disease type. The BMI association was not significant in the parsimonious, sex-adjusted, multiple-imputation, or continuous-outcome analyses and should not be regarded as a robust predictor. A patient with higher BMI may meet the 5% threshold while remaining well nourished, whereas a smaller loss in a lean patient may be clinically important. The borderline age finding was not interpreted because the analyses were exploratory and no multiplicity correction was applied.

Differences between lymphoma and multiple myeloma should also be interpreted cautiously. Patients with myeloma were older and had a higher BMI, whereas patients with lymphoma had greater measured body-weight reduction and more frequent TPN use. These differences may reflect conditioning regimen, prior lines of therapy, corticosteroid exposure, disease status, infection, mucositis severity, neutropenia duration, nutritional intake, renal function, or supportive-care practices. These variables were unavailable or incomplete; causal attribution to diagnosis is therefore not justified.

This study has several limitations. Its retrospective, single-center design may limit generalizability. Admission and discharge weights were not collected under a verified study-specific protocol, and detailed fluid balance, edema, diuretic use, hydration status, contemporaneous renal function, and serial standardized weights were unavailable. Body weight cannot distinguish adipose tissue, muscle, and fluid change; body-composition measurements and detailed energy and protein intake were also unavailable. Nutritional support was not randomized and its timing was unknown. Laboratory measurements were obtained within a broad ± 10-day window rather than serially. The primary model had eight predictors but only 37 events among 92 complete cases (4.6 events per predictor), creating overfitting and selection concerns. Firth, parsimonious, sex-adjusted, continuous-outcome, and multiple-imputation analyses do not remove these limitations, and the missing-at-random assumption could not be verified. Residual non-normality in the continuous model required robust standard errors. Important transplant-related confounders were not captured. Finally, the ≥5% threshold was pragmatic and is not a validated short-term malnutrition definition.

Future studies should incorporate standardized serial weights, prospective fluid-balance and renal-function data, repeated nutritional screening, body-composition measures, detailed energy and protein intake, and patient-reported symptoms. Conditioning regimen, prior therapy, corticosteroid exposure, disease status, infection, neutropenia duration, mucositis severity, and timing of supportive care should also be recorded.

Strengths include a clinically relevant mixed ASCT cohort and integration of nutritional, laboratory, symptom, supportive-care, and measured body-weight data within the same cohort.

## 5. Conclusions

Nutritional vulnerability during ASCT was characterized by frequent reductions in measured body weight and a high prevalence of nutrition-impact symptoms. These scale-weight changes cannot be equated with tissue loss because detailed fluid-related data were unavailable. Admission NRS-2002 screening and subsequent acute weight change are related but distinct assessments, and the lack of a significant association is inconclusive. The diagnosis association may reflect unmeasured transplant-related differences, while the BMI association was not robust. All multivariable findings remain exploratory.

## Figures and Tables

**Table 1:** Missingness of principal analysis variables.

Variable	Available, n/N (%)	Missing, n/N (%)
Measured body-weight change	131/131 (100%)	0/131 (0%)
Age, diagnosis, sex, admission BMI	131/131 (100%)	0/131 (0%)
Admission NRS-2002 score	131/131 (100%)	0/131 (0%)
Albumin	123/131 (94%)	8/131 (6%)
CRP	100/131 (76%)	31/131 (24%)
NLR	99/131 (76%)	32/131 (24%)
Diarrhea	130/131 (99%)	1/131 (1%)
Anorexia	130/131 (99%)	1/131 (1%)
ONS	130/131 (99%)	1/131 (1%)
TPN	129/131 (98%)	2/131 (2%)

Laboratory missingness reflected values unavailable within the prespecified retrospective extraction window. CRP, C-reactive protein; NLR, neutrophil-to-lymphocyte ratio; ONS, oral nutritional supplement; TPN, total parenteral nutrition.

**Table 2:** Cohort characteristics by diagnosis.

Characteristic	Overall (*n* = 131)	Lymphoma (*n* = 74)	Myeloma (*n* = 57)	*p*-Value
Age, years	54 ± 14	50 ± 15	60 ± 10	<0.001
Female sex	65/131 (50%)	39/74 (53%)	26/57 (46%)	0.42
Length of stay, days	24 (20–29)	26 (21–30)	22 (20–27)	0.06
Admission BMI, kg/m^2^	27 ± 5	26 ± 5	28 ± 5	0.02
Obesity, BMI ≥ 30	31/131 (24%)	14/74 (19%)	17/57 (30%)	0.15
≥5% measured body-weight reduction, n/N (%)	53/131 (40%)	38/74 (51%)	15/57 (26%)	0.004
Measured body-weight reduction, %	4 (2–6)	5 (3–7)	3 (1–5)	0.003
NRS-2002 score	2 (2–2.5)	2 (2–2.8)	2 (2–2)	0.68
NRS-2002 ≥ 3	33/131 (25%)	19/74 (26%)	14/57 (25%)	0.88
Albumin, g/L	39 ± 4	40 ± 4	39 ± 4	0.59
CRP, mg/L	2.2 (1.1–5.3)	3.5 (1.1–6.2)	1.7 (1.1–4.7)	0.12
NLR	16.5 (10.0–36.5)	12.6 (7.2–17.1)	36 (19.8–54.5)	<0.001
ONS support	63/130 (48%)	37/73 (51%)	26/57 (46%)	0.57
TPN support	67/129 (52%)	44/72 (61%)	23/57 (40%)	0.02
Mucositis	28/130 (22%)	17/73 (23%)	11/57 (19%)	0.58
Diarrhea	84/130 (65%)	48/73 (66%)	36/57 (63%)	0.76
Anorexia	84/130 (65%)	42/73 (58%)	42/57 (74%)	0.06

Data are mean ± SD, median (IQR), or n/N (%). BMI, body mass index; CRP, C-reactive protein; NLR, neutrophil-to-lymphocyte ratio; NRS-2002, Nutritional Risk Screening 2002; ONS, oral nutritional supplement; TPN, total parenteral nutrition.

**Table 3:** Nutritional and inflammatory characteristics according to NRS-2002 risk category.

Variable	NRS-2002 < 3 (*n* = 98)	NRS-2002 ≥ 3 (*n* = 33)	*p*-Value
Albumin, g/L, median (IQR)	40.1 (37.6–41.8)	39.3 (36.9–41.1)	0.33
CRP, mg/L, median (IQR)	2.2 (1.0–5.1)	3.3 (1.4–8.6)	0.14
NLR, median (IQR)	19 (10–36.5)	16 (10.7–32)	0.84
Measured body-weight reduction, %, median (IQR)	4 (3–7)	4 (2–6)	0.18
Length of stay, days, median (IQR)	24 (21–29)	22 (15–33)	0.39
Diarrhea, n/N (%)	63/98 (64%)	21/32 (66%)	1.00
Anorexia, n/N (%)	64/98 (65%)	20/32 (63%)	0.83
TPN, n/N (%)	50/97 (52%)	17/32 (53%)	1.00

Data are shown for NRS-2002 < 3 (*n* = 98) and NRS-2002 ≥ 3 (*n* = 33); denominators vary across variables because of missing data. CRP, C-reactive protein; NLR, neutrophil-to-lymphocyte ratio; TPN, total parenteral nutrition.

**Table 4:** Exploratory multivariable logistic regression for meeting the ≥5% measured body-weight reduction threshold.

Predictor	OR	95% CI	*p*-Value
Age, per year	0.95	0.91–1.00	0.046
Multiple myeloma vs. lymphoma	0.20	0.07–0.56	0.002
NRS-2002 ≥ 3	1.09	0.34–3.47	0.89
Admission BMI, per kg/m^2^	1.12	1.01–1.24	0.03
Albumin, per g/L	0.91	0.80–1.04	0.16
CRP, per mg/L	1.00	0.98–1.03	0.90
Diarrhea	0.93	0.34–2.58	0.90
Anorexia	1.84	0.63–5.39	0.27

Complete-case *n* = 92: 37 events and 55 non-events (4.6 events per predictor). AUC = 0.77; Brier score = 0.19; Hosmer–Lemeshow χ^2^ = 12.3 (8 df), *p* = 0.14; VIF range, 1.05–1.32. These are apparent-sample diagnostics without internal validation. OR, odds ratio; CI, confidence interval; BMI, body mass index.

**Table 5:** Multivariable linear regression of continuous measured body-weight reduction.

Predictor	β, Percentage Points	95% CI	*p*-Value
Age, per year	−0.05	−0.13 to 0.03	0.21
Multiple myeloma vs. lymphoma	−1.9	−3.1 to −0.56	0.005
NRS-2002 ≥ 3	−0.26	−1.8 to 1.3	0.74
Admission BMI, per kg/m^2^	0.12	−0.001 to 0.24	0.051
Albumin, per g/L	0.01	−0.18 to 0.20	0.92
CRP, per mg/L	0.05	−0.05 to 0.14	0.34
Diarrhea	0.11	−1.29 to 1.52	0.88
Anorexia	−0.04	−1.35 to 1.27	0.95

Complete-case *n* = 92. Positive β values indicate greater measured body-weight reduction. HC3 robust standard errors were used. R^2^ = 0.23; adjusted R^2^ = 0.16.

## Data Availability

The data that support the findings of this study are available from the corresponding author upon reasonable request, subject to institutional and ethical restrictions.
